# Improving Between-Group Effect Size for Multi-Site Functional Connectivity Data via Site-Wise De-Meaning

**DOI:** 10.3389/fncom.2021.762781

**Published:** 2021-12-02

**Authors:** Alexandra M. Reardon, Kaiming Li, Xiaoping P. Hu

**Affiliations:** ^1^Department of Bioengineering, University of California, Riverside, Riverside, CA, United States; ^2^Center for Advanced Neuroimaging, University of California, Riverside, Riverside, CA, United States

**Keywords:** autism spectrum disorder, effect size, functional connectivity, multi-site, resting-state functional MRI, Schizophrenia

## Abstract

**Background:** Multi-site functional MRI (fMRI) databases are becoming increasingly prevalent in the study of neurodevelopmental and psychiatric disorders. However, multi-site databases are known to introduce site effects that may confound neurobiological and measures such as functional connectivity (FC). Although studies have been conducted to mitigate site effects, these methods often result in reduced effect size in FC comparisons between controls and patients.

**Methods:** We present a site-wise de-meaning (SWD) strategy in multi-site FC analysis and compare its performance with two common site-effect mitigation methods, i.e., generalized linear model (GLM) and Combining Batches (ComBat) Harmonization. For SWD, after FC was calculated and Fisher z-transformed, the site-wise FC mean was removed from each subject before group-level statistical analysis. The above methods were tested on two multi-site psychiatric consortiums [Autism Brain Imaging Data Exchange (ABIDE) and Bipolar and Schizophrenia Network on Intermediate Phenotypes (B-SNIP)]. Preservation of consistent FC alterations in patients were evaluated for each method through the effect sizes (Hedge’s g) of patients vs. controls.

**Results:** For the B-SNIP dataset, SWD improved the effect size between schizophrenic and control subjects by 4.5–7.9%, while GLM and ComBat decreased the effect size by 22.5–42.6%. For the ABIDE dataset, SWD improved the effect size between autistic and control subjects by 2.9–5.3%, while GLM and ComBat decreased the effect size by up to 11.4%.

**Conclusion:** Compared to the original data and commonly used methods, the SWD method demonstrated superior performance in preserving the effect size in FC features associated with disorders.

## Introduction

Neuroimaging has become a powerful tool in studying psychiatric disorders (Peter et al., 2018). Functional magnetic resonance imaging (fMRI) allows for the study of aberrant functional connectivity (FC), predictions of normal individuals vs. patients, early identification of neurological diseases, neuromarkers, and responses to treatment (Van Horn and Toga, 2009). Many traditional fMRI studies were limited by statistical power, since large-scale data is difficult to obtain at a single imaging site due to limited diseased population in one geographical location, limited time, and limited funds (Van Horn and Toga, 2009). Multi-site neuroimaging consortiums are becoming increasingly common in attempts to capture heterogeneity associated with various disorders, as well as to increase geographic variability, sample size, and statistical power (Van Horn and Toga, 2009).

While there are many benefits to multi-site consortiums, there are significant challenges in combining the data for analysis. FMRI data from different sites may contain scanner and site variability, leading to conflicting results and inferior reliability (Van Horn and Toga, 2009; Newton et al., 2012; Birn et al., 2013; Rane et al., 2014; An et al., 2017; Badhwar et al., 2019). Scanner variability can arise from different scanning vendors, scanner technology, and field inhomogeneities (Van Horn and Toga, 2009). Sites with the same scanner vendors and models have been found to introduce different field inhomogeneities that have affected the way the data was interpreted (Van Horn and Toga, 2009). Additionally, different scanner manufacturers are known to have different levels of test-retest reliability. It has been reported that Siemen’s scanners have better consistency than Philips’s scanners (An et al., 2017; Badhwar et al., 2019). In many multi-site consortiums, individual imaging sites utilized different scanning parameters, including repetition time, echo time, acquisition time, voxel size, flip angle, field of view, and slice thickness, in collecting fMRI data. The use of different scanning parameters has been known to influence resting-state fMRI results (Newton et al., 2012; Birn et al., 2013; Rane et al., 2014). For example, increasing the acquisition time of scans from 5 to 13 min has been proven to improve the reliability and similarity of functional correlations in resting state scans (Birn et al., 2013). Newton et al. (2012) reported that decreasing voxel size dimensions increased FC correlations in resting state scans, while Rane et al. (2014) demonstrated that a short TE (TE = 15 ms) in scans led to results less correlated with group results than scans acquired at a higher TE (TE = 35 ms and TE = 55 ms).

Efforts to reduce site variability have been made through homogenizing scanning protocols and/or through site-to-site quality assurance via standardized brain imaging phantoms (Yu et al., 2018). While these methods mitigate some of the variability associated with site-effects, in existing multi-site consortiums where data were not originally purposed for aggregation, homogenized scanning protocols and imaging phantoms were not available. One study quantified the sampling bias and engineering measurement bias of a traveling subject cohort who received resting-state scans at multiple imaging sites (Yamashita et al., 2019). This method was able to remove only the measurement bias, therefore improving signal-to-noise ratio. However, utilizing a traveling-cohort is costly, time consuming, and may be impractical with many established multi-site consortiums, and therefore a post-acquisition method to mitigate site-effects is desirable.

Attempts to reduce multi-site consortium variability in FC analysis of fMRI data after acquisition include generalized linear model (GLM) and Combining Batches (ComBat) harmonization (Rao and Joao, 2017; Yu et al., 2018; Yamashita et al., 2019). GLM modifies FC values to account for site differences, but important FC features associated with patient groups may be compromised after this method (Rao and Joao, 2017; Yamashita et al., 2019). ComBat utilizes site-specific scaling factors and an empirical Bayesian criterion to shift samples to the grand mean and pooled variance across sites (Yu et al., 2018). It has demonstrated effectiveness in small samples of resting-state fMRI data using homogenized scanning parameters. However, it is unclear if ComBat harmonized fMRI data preserves the functional networks associated with psychiatric disorders or can accurately account for FC effects imposed by heterogeneous scan parameters (Yu et al., 2018). ComBat also centers the FC data of each site to the overall, grand mean of all samples, thus resulting in harmonized FC features that lose their original physical meaning (Da-Ano et al., 2020).

Although some multi-site consortiums may use phantoms or homogenous scanning parameters, there is always the possibility that sites will decide to aggregate FC data after image acquisition. There is a great need for a site-effect mitigation method that can be applied after acquisition, on heterogeneous scanning parameters, and that preserves functional networks associated with psychiatric disorders. Examples of such multi-site database are the Autism Brain Imaging Data Exchange (ABIDE) and the Bipolar and Schizophrenia Network on Intermediate Phenotypes (B-SNIP) (Tamminga et al., 2013; Di Martino et al., 2014). ABIDE is a consortium of neuroimaging data from Autism Spectrum Disorder (ASD) subjects and healthy controls (HC) from 17 international sites, while B-SNIP is a consortium of Schizophrenia (SZ), Schizoaffective disorder (SA), Bipolar disorder subjects and HCs from 5 different imaging sites (Tamminga et al., 2013). Both datasets include sites that utilize different resting-state scanning parameters, protocols, and scanner models.

Here, we describe a site-wise de-meaning (SWD) strategy for multi-site FC analysis of fMRI data and compare its performance with two common site-effect mitigation methods (GLM, and ComBat Harmonization). We (1) establish the consistent FC differences between disease groups and control groups in literature, (2) apply site-effect mitigation methods (GLM, ComBat, SWD) to multi-site FC data, and (3) compare the effect size of established FC findings of the three site-effect mitigation methods.

## Materials and Methods

### Datasets

#### Bipolar and Schizophrenia Network on Intermediate Phenotypes

Resting-state fMRI data of 317 subjects from 4 sites in B-SNIP with a Diagnostic and Statistical Manual, 4th Edition (DSM-IV) SZ diagnosis (*n* = 149) and the corresponding HCs (*n* = 168) were included in this study (Tamminga et al., 2013). Demographic information including site, sample size, sex, and age is shown in Table 1.

**TABLE 1:** Demographic information for the SZ and HC subjects from B-SNIP.

**Site**	**N**	**% SZ**	**% Male**	**Mean age ± std (yr)**
Baltimore	188	71.3	52.8	38.7 ± 12.7
Boston	52	65.4	50.0	34.7 ± 11.5
Dallas	143	59.4	44.1	39.6 ± 11.4
Hartford	129	705	51.9	33.9 ± 11.3

#### Autism Brain Imaging Data Exchange

Resting-state fMRI data from 850 subjects in ABIDE I with an ASD DSM-IV-TR diagnosis (*n* = 355) and the corresponding HCs (*n* = 495) from the same sites were used in this study (First and Gibbon, 2004; Di Martino et al., 2014). Demographic information including site, sample size, sex, age, and mean Full Scale IQ (FIQ) is shown in Table 2.

**TABLE 2:** Demographic information for the ASD and HC subjects from ABIDE.

**Site**	**N**	**% ASD**	**% Male**	**Age Mean ± std**
Caltech	23	47.8	78.3	27.1 ± 5.8
CMU	32	40.6	78.1	26.8 ± 9.8
KKI	44	25.0	77.3	10.1 ± 1.2
Leuven	62	46.8	88.7	18.1 ± 5.0
Ludwig	34	5.9	88.2	25.3 ± 10.3
NYU	122	43.4	73.0	13.8 ± 5.8
Olin	36	55.6	86.1	16.8 ± 3.5
SBL	17	11.8	100	32.7 ± 7.0
SDSU	24	12.5	70.8	14.1 ± 1.9
Trinity	35	28.6	100	16.8 ± 3.5
UCLA	102	54.9	88.2	13.1 ± 2.5
UMich	129	41.1	81.4	14.2 ± 3.3
UPitt	56	51.8	85.7	18.8 ± 6.9
USM	100	57.0	100	22.1 ± 7.7
Yale	34	17.7	70.6	13.1 ± 2.8

*Caltech, California Institute of Technology; CMU, Carnegie Mellon University; FIQ, Full Scale IQ; KKI, Kennedy Krieger Institute; Ludwig, Ludwig Maximilians University Munich; NYU, New York University Langone Medical Center; Olin, Olin, Institute of Living at Hartford Hospital; SDSU, San Diego State University; SBL, Social Brain Lab; Trinity, Trinity Centre for Health Sciences; UCLA, University of California, Los Angeles; Leuven, University of Leuven; UMich, University of Michigan; UPitt, University of Pittsburgh School of Medicine; USM, University of Utah School of Medicine and Yale, Yale Child Study Center.*

### Image Acquisition

Imaging data used in this analysis were collected on 3T MRI scanners. Scan parameters for the resting-state fMRI protocols from B-SNIP are summarized in Table 3 and scan parameters from ABIDE are summarized in Table 4. For each subject, a T_1_-weighted structural image was collected and used for registration to the MNI152 space. Full details for acquisition parameters, informed consent, and site-specific protocols can be found at http://fcon_1000.projects.nitrc.org/indi/abide/abide_I.html for ABIDE and http://b-snip.org/for B-SNIP (Tamminga et al., 2013; Di Martino et al., 2014).

**TABLE 3:** Resting-state fMRI scan parameters for subjects in B-SNIP.

**Site**	**Scanner**	**TR (ms)**	**TE (ms)**	**Acq. time**	**Voxel size (mm^2^)**	**Number of slices**	**Flip angle (degree)**
Baltimore	Siemens Trio Tim	2,210	30	5 min	3.4x3.4x3	36	70
Boston	GE Signa HDX	3,000	27	5 min	3.4x3.4x4	30	60
Dallas	Philips	1,500	27	5 min	3.4x3.4x4	29	60
Hartford	Siemens Allegra	1,500	27	5 min	3.4x3.4x5	29	70

*Acquisition Time (Acq. Time), Echo Time (TE), Repetition Time (TR).*

**TABLE 4:** Resting-state fMRI scan parameters for subjects in ABIDE.

**Site**	**Scanner**	**TR (ms)**	**TE (ms)**	**Acq. time (min)**	**Voxel size (mm)**	**Number of slices**	**Flip angle (deg)**
Caltech	Siemens Trio	2,000	30	5:04	3.5 × 3.5 × 3.5	34	75
CMU	Siemens Verio	2,000	30	8:06	3 × 3 × 3	28	73
KKI	Philips Achieva	2,500	30	6:40	3.59 × 3.59 × 4	47	75
Leuven	Philips Intera	1,667	33	7:06	3 × 3 × 4	32	90
Ludwig	Siemens Verio	3,000	30	6:06	3 × 3 × 4	28	80
NYU	Siemens Allegra	2,000	15	6:00	3.75 × 3.75 × 3.8	33	90
Olin	Siemens Allegra	1,500	27	5:15	2.75 × 2.75 × 2.72	29	60
SBL	Philips Intera	2,200	30	7:28	3.44 × 3.44 × 3.4	38	80
SDSU	GE MR750	2,000	30	6:10	3.13 × 3.13 × 4.5	34	90
Trinity	Philips Achieva	2,000	28	5:06	3 × 3 × 3.5	38	90
UCLA	Siemens Trio	3,000	28	6:06	3 × 3 × 4	34	90
UMich	GE Signa	2,000	30	10:00	3.44 × 3.44 × 3	40	90
UPitt	Siemens Allegra	1,500	25	5:06	3.1 × 3.1 × 4	29	70
USM	Siemens Trio	2,000	28	8:06	3.4 × 3.4 × 3	40	90
Yale	Siemens Trio	2,000	25	6:50	3.4 × 3.4 × 4	34	60

*Acq, Time, Acquisition time; Caltech, California Institute of Technology; CMU, Carnegie Mellon University; deg, degree; TE, Echo time; FOV, Field of view; KKI, Kennedy Krieger Institute; Ludwig, Ludwig Maximilians University Munich; NYU, New York University Langone Medical Center; Olin, Olin, Institute of Living at Hartford Hospital; TR, Repetition time; SDSU, San Diego State University; SBL, Social Brain Lab; Trinity, Trinity Centre for Health Sciences; UCLA, University of California, Los Angeles; Leuven, University of Leuven; UMich, University of Michigan; UPitt, University of Pittsburgh School of Medicine; USM, University of Utah School of Medicine; Yale, Yale Child Study Center.*

### Preprocessing

The resting-state fMRI data was preprocessed using the Connectome Computation System pipeline (Zuo et al., 2013). Steps included slice time correction, motion correction, skull stripping, global mean intensity normalization, nuisance signal regression, band pass filtering (0.01–0.1 Hz), and registration of the resting-state fMRI image to the T1-weighted image, followed by a transformation to standard space (Zuo et al., 2013). The resting state fMRI data was then parcellated into 200 regions of interest (ROIs) using a spatially constrained spectral clustering algorithm based on functional parcellations by Cameron Craddock (Craddock et al., 2012).

### Functional Connectivity Matrices and Parcellation

Pearson’s correlation coefficient was used to ascertain the FC of each region of interest (ROI) pair, resulting in a 200 × 200 FC matrix for each subject. Each correlation coefficient was Fisher z-transformed, then linear regression was used to regress out age and sex covariates to ensure that these confounding variables did not affect results.

### Established Functional Connectivity Differences in Diagnostic Groups

The most common resting-state FC findings between disease and control groups was determined by performing a literature review in PubMed to identify relevant studies published within the last 15 years for FC differences between SZ and HC (keywords: SZ, FC, resting-state, functional MRI) and for FC differences between ASD and HC (keywords: ASD, FC, resting-state, functional MRI).

### Comparison of Methods

We compared the effect size of FC differences between patients and controls for the following methods: (1) GLM, (2) ComBat (Johnson et al., 2007), and (3) SWD.

(1) **GLM**

After Fisher z-transforming the FC data, multiple linear regression with terms for age, sex, and site was performed in MATLAB. The regression model can be written as:


$${\text{y}}_{\text{ijv}}=\alpha_{\text{v}}+{\text{X}}_{\text{ij}}^{\text{T}}\,\beta_{\text{v}}+\varepsilon_{i\,j\,v}$$

Where y_ijv_ represents the connectivity at every site (i), subject (j), and for every ROI pair (v), α_v_ is the average connectivity value for a particular connectivity value (v), ${\text{X}}_{\text{ij}}^{\text{T}}$ is the design matrix for the covariates (age, sex, site) for every site (i), and subject (j), and β_v_ is the vector of regression coefficients corresponding to ${\text{X}}_{\text{ij}}^{\text{T}}$. The removal of site-effects is done by subtracting the estimated site-effects:


$${\text{y}}_{\text{ijv}}^{\text{GLM}}={\text{y}}_{\text{ijv}}-\alpha_{\text{v}}-{\text{X}}_{\text{ij}}^{\text{T}}\,\beta_{\text{v}}$$

(2) **ComBat**

FC values were Fisher z-transformed and a multivariate linear mixed effects regression with terms for biological variables and scanner were used to model FC (Yu et al., 2018). The ComBat harmonization model can be written as:


$${\text{y}}_{\text{ijv}}=\alpha_{\text{v}}+{\text{X}}_{\text{ij}}^{\text{T}}\,\beta_{\text{v}}+\gamma_{\text{iv}}+\delta_{\text{iv}}\,\varepsilon_{\text{ijv}}$$

Where y_ijv_ represents the connectivity at every site (i), subject (j), and for every ROI pair (v), α_v_ is the average connectivity value for a particular connectivity value (v), X^*T*^_*ij*_ is a design matrix for the covariates of interest (age, sex, and diagnostic group) for every subject (j), β_v_ is a vector of regression coefficients corresponding to X^*T*^_*ij*_, γ_iv_ and δ_iv_ are the additive (or location parameter) and multiplicative (or scale parameter), respectively, of site-effects of site i for connectivity value v (Yu et al., 2018). ComBat was performed in MATLAB and the adjusted FC values are given by:


$${\text{y}}_{\text{ijv}}^{\text{ComBat}}=\frac{{\text{y}}_{\text{ijv}}-\hat{\alpha_{\text{v}}}-{\text{X}}_{\text{ij}}^{\text{T}}\,\hat{\beta_{\text{v}}}-\gamma_{\text{iv}}^{*}}{\delta_{\text{iv}}^{*}}+\hat{\alpha_{\text{v}}}+{\text{X}}_{\text{ij}}^{\text{T}}\,\hat{\beta_{\text{v}}}$$

where $\gamma_{\text{iv}}^{*}$ and $\delta_{\text{iv}}^{*}$ are the empirical Bayes estimate of the additive (or location parameter) and multiplicative (or scale parameter), respectively, of site-effects of site i for connectivity value v (Yu et al., 2018). Age and sex effects were then regressed out of the ComBat harmonized FC data.

(3) ***SWD***

The Fisher z-transformed data with age and sex regressed out is referred to in Algorithm as FC. A single overall mean value for each site was determined by averaging all FC values for every subject in each site. The mean value was then subtracted from each FC feature for every subject.

**Algorithm:** Site-wise de-meaning (SWD).

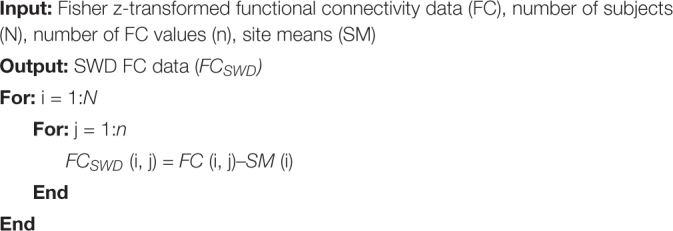

### Effect Size

To evaluate how each method affects the underlying neurobiological measures, Hedge’s g was used to calculate the effect size of consistent FC alterations in group analysis (ASD vs. HC and SZ vs. HC) for (1) original data with sex and age regressed out, (2) GLM, (3) ComBat harmonized data, and (4) SWD. It is suggested that 0.2 is considered to be a small effect size, 0.5 represents a medium effect size and 0.8 represents a large effect size (Hedges and Olkin, 1985). The following equation was used to calculate Hedge’s g:


Hedge⁢s′⁢g=MH⁢C-MA⁢S⁢DSDp⁢o⁢o⁢l⁢e⁢d*

where M_*HC*_ is the mean connectivity of HCs for a particular connectivity value, M_*ASD*_ is the mean connectivity of ASDs for a particular connectivity value, and SD^∗^_*pooled*_ is the weighted and pooled standard deviation.

### Ethics

Ethical guidelines used in this manuscript are available at http://fcon_1000.projects.nitrc.org/indi/abide/abide_I.html for ABIDE and http://b-snip.org/for B-SNIP (Tamminga et al., 2013; Di Martino et al., 2014).

## Results

### Consistent Functional Connectivity Alterations

#### Healthy Controls vs. Schizophrenia Literature Review Findings

**FIGURE 1:**
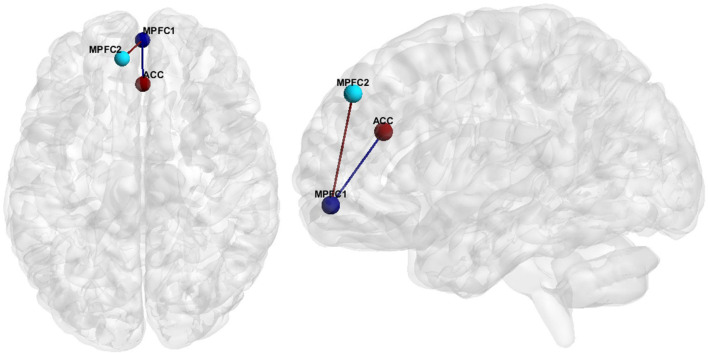
FC features associated with SZ. Medial Prefrontal Cortex (MPFC), Anterior Cingulate Cortex (ACC).

Hypoconnectivity, specifically in the medial prefrontal cortex (MPFC), as well as between the MPFC and the anterior cingulate cortex (ACC) (Figure 1), was the most common resting state finding in SZ. Further information regarding the literature findings on hypoconnectivity within MPFC and between MPFC and ACC can be found in Tables 5, 6, respectively.

**TABLE 5:** Resting state fMRI studies finding within MPFC hypoconnectivity in SZ participants.

**Author**	**n (HC/SZ)**	**Age mean (std) (HC/SZ)**	**N female (HC/SZ)**	**Analysis method**	**B-SNIP**	**SZ participant info**
Bluhm et al. (2007)	17/17	30.94 (12.60)/ 33.54 (13.77)	3/3	Seed	No	15 paranoid SZ/2 undifferentiated SZ
Chen et al. (2017)	20/20	41.6 (13.6)/ 40.3 (13.8)	13/11	Local FCD	No	SZ only
Cole et al. (2011)	22/23	37.18 (7.59)/ 36.54 (9.36)	6/5	Seed	No	SZ only
Du et al. (2016)	82/82	37.7 (10.8)/ 38.0 (14.0)	19/17	ROI	No	SZ only
Fang et al. (2018)	22/20	24.3 (4.8)/ 24.2 (4.8)	10/13	Seed/ROI (Effective connectivity)	No	FES
Guo et al. (2014)	50/49	23.48 (2.49)/ 22.69 (4.62)	27/19	Network homogeneity	No	SZ only
He et al. (2013)	113/115	26.61 (8.9)/ 25.36 (8.2)	56/62	fALFF	No	FES
Huang et al. (2010)	66/66	24.5 (8.6)/ 24.2 (8.4)	36/36	ALFF	No	FES (Treatment naïve)
Lui et al. (2010)	34/34	25.0 (8.0)/ 24.6 (8.5)	21/21	ICA	No	FES (Treatment naïve)
Meda et al. (2012)	324/296	35.2 (13.4)/ 34.9 (12.2)	144/97	ICA	Yes	SZ only
Mingoia et al. (2012)	25/25	29.1 (8.6)/ 30 (7.3)	10/8	pICA	No	SZ only
Mwansisya et al. (2013)	33/41	24.52 (6.33)/ 23.88 (5.85)	17/16	Seed	No	FES
Ongür et al. (2010)	15/14	37.9 (9.5)/ 42.3 (9.5)	6/6	ICA	No	SZ and SA
Su et al. (2013)	25/25	42.5 (9.9)/ 42.5 (9.9)	13/13	Seed	No	SZ only

*ALFF, Amplitude of Low Frequency Fluctuations; BOLD, Blood Oxygenation Level Dependent; FES, First Episode Schizophrenia; fALFF, Fractional Amplitude of Low Frequency Fluctuations; FCD, Functional Connectivity Density; ICA, Independent Component Analysis; pICA, Probabilistic ICA.*

**TABLE 6:** Resting state fMRI studies finding MPFC to ACC hypoconnectivity in SZ participants.

**Author**	**n (HC/SZ)**	**Age mean (std) (HC/SZ)**	**N Female (HC/ASD)**	**Analysis method**	**B-SNIP**	**SZ participant info**
Alonso-Solís et al. (2015)	20/19	37.75 (7.4)/ 40.05 (8.9)	7/6	Seed	No	Auditory hallucinating SZ participants
Anticevic et al. (2015)	56/73	31.25 (10.3)/ 32.99 (10.9)	32/24	Seed	No	SZ only
Camchong et al. (2009)	29/29	41.1 (10.6)/ 41.3 (9.28)	11/11	ICA/ROI	No	SZ only
Fang et al. (2018)	22/20	24.3 (4.8)/ 24.2 (4.8)	10/13	Seed/ROI (Effective connectivity)	No	FES
Holt et al. (2011)	17/18	40 (12.5)/ 35.9 (13.7)	6/6	Seed	No	SZ only
Hoptman et al. (2014)	31/33	38.6 (9)/ 38.2 (10.4)	9/6	Seed	No	SZ and SA
Jang et al. (2011)	16/16	22.06 (1.65)/ 21.32 (5.65)	7/7	Seed	No	Genetic high risk for SZ
Kyriakopoulos et al. (2012)	20/25	16.3 (2.1)/ 16.1 (2.5)	8/11	Seed	No	EOS
Li et al. (2019)	2567/2588	31.17/31	1168/1092	ICA	No	SZ only Meta-Analysis
Lui et al. (2015)	59/37	38 (17)/ 36 (14)	33/15	ALFF	Yes	SZ only
Meda et al. (2014)	324/296	35.2 (13.4)/ 34.9 (12.2)	144/97	ICA	Yes	SZ only
Penner et al. (2016)	24/24	23.8 (4.3)/ 23.2 (4.2)	12/3	Seed	No	SZ only
Zhou et al. (2015)	10/91	33.3 (10.5)/ 33.9 (7.7)	55/40	Seed	No	SZ only

*ALFF, Amplitude Low Frequency Fluctuations; EOS, Early onset Schizophrenia; FES, First Episode Schizophrenia; ICA, Independent Component Analysis; SA, Schizoaffective Disorder; SZ, Schizophrenia.*

#### Autism Spectrum Disorder Findings

**FIGURE 2:**
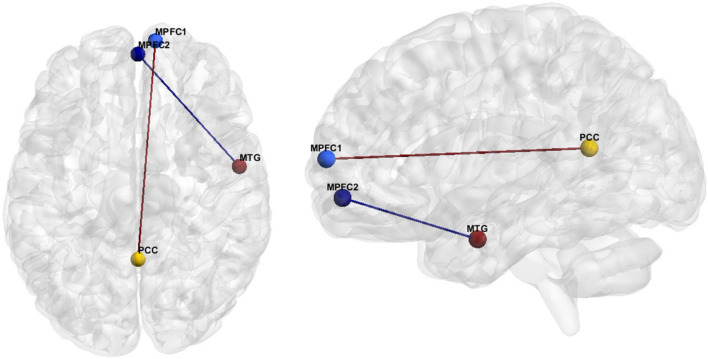
FC features associated with ASD. Medial prefrontal cortex (MPFC), Posterior Cingulate Cortex (PCC), Medial Temporal Gyrus (MTG).

The hypoconnectivity hypothesis of ASD posits that behavioral features of ASD arise from reduced neural connections in the brain (Just et al., 2012). The most common resting state fMRI finding regarding ASD FC was anterior-posterior DMN hypoconnectivity (see Hull et al., 2016). More specifically, our literature review resulted in eighteen studies reporting hypoconnectivity between the posterior cingulate cortex (PCC)/precuneus and the MPFC (Figure 2 and Table 7). Hypoconnectivity between the MPFC in the frontal lobe and MTG of the temporal lobe was the second most common finding in the ASD literature (Figure 2 and Table 8).

**TABLE 7:** Resting state fMRI studies of the primary FC feature of ASD finding MPFC to PCC hypoconnectivity compared to HCs.

**Author**	**N (HC/ASD)**	**Age mean (std) (HC/ASD)**	**N female (HC/ASD)**	**Analysis method**	**ABIDE**	**ASD participant info**
Abbott et al. (2016)	38/37	13.0 (2.6)/ 13.9 (2.6)	8/5	iFC	No	ASD only
Assaf et al. (2010)	15/15	17.1 (3.6)/ 15.7 (3.0)	2/1	ICA	No	HFA
Cherkassky et al. (2006)	57/57	24 (9.0)/ 24 (10.6)	5/4	ROI	No	HFA
Doyle-Thomas et al. (2015)	44/71	12.2 (3.8)/ 12.3 (3.1)	0/0	Seed	No	ASD
Eilam-Stock et al. (2014)	15/17	27.1 (8.2)/ 26.1 (6.5)	NA	Seed	No	HFA (12), ASP (5)
Falahpour et al. (2016)	76/76	64 (12)/ 62 (14)	12/9	iFC	Yes	ASD
Falahpour et al. (2016)	32/32	13.5 (2.7)/ 14.3 (2.4)	5/4	SD-iFC	No	ASD
Joshi et al. (2017)	16/15	21.9 (3.5)/ 21.6 (3.7)	0/0	Seed	No	HFA
Jung et al. (2014)	21/19	24.8 (4.3)/ 25.3 (6.9)	0/0	Seed	No	HFA
Jones et al. (2010)	20/17	17.1 (2.1)/ 16.1 (2.6)	0/0	Seed	No	HFA
Lee et al. (2016)	517/458	16. 5 (7.3)/16.2 (7.4)	90/54	FCD	Yes	ASD/ASP/PDD-NOS
Liu et al. (2020)	548/506	16.86 (7.55)/ 16.59 (8.05)	95/60	Seed	Yes	ASD/ASP/PDD-NOS
Long et al. (2016)	64/64	Child Cohort: 9.3 (1.5)/9.6 (1.0) Adolescent Cohort: 14.5 (1.9)/13.7 (1.8) Adult Cohort: 25.5 (4.2)/ 25.4 (5.9)	10/10	Seed	Yes	ASD/ASP/PDD-NOS
Maximo et al. (2013)	29/29	13.5 (2.2)/ 13.8 (2.4)	7/4	ReHo	No	HFA
Monk et al. (2009)	12/12	27 (6.1)/ 26 (5.9)	2/1	Seed	No	ASD (7)/ASP (3) and PDD-NOS (3)
Murdaugh et al. (2012)	14/13	22.6 (4.2)/ 21.4 (3.9)	0/0	Seed	No	HFA
Washington et al. (2014)	24/24	10.08 (3.17)/ 10.88 (2.27)	3/3	ICA/ROI	No	ASD only
Weng et al. (2010)	15/16	16 (1.44)/ 15 (1.45)	2/1	Seed	No	ASP (2), PDD-NOS (8), ASD (6)
Yerys et al. (2015)	22/22	11.37 (1.56)/ 11.41 (1.51)	4/4	Seed	No	ASD

*ASP, Asperger’s; FCD, Functional Connectivity Density; HFA, High Functioning Autism; ICA, Independent Component Analysis; iFC, Intrinsic Functional Connectivity; PDD-NOS, Pervasive Development Disorder-Not Otherwise Specified; ROI, Region of Interest; ReHo, Regional Homogeneity; SD-iFC, Standard deviation of the sliding window correlation.*

**TABLE 8:** Resting state fMRI studies for the secondary FC feature of ASD finding MPFC—MTG hypoconnectivity for ASD participants.

**Author**	**N (HC/ASD)**	**Age mean (std) (HC/ASD)**	**N female (HC/ASD)**	**Analysis Method**	**ABIDE**	**ASD participant info**
Borràs-Ferrís et al. (2019)	74/74	Child Cohort 10.63 (0.86) Adolescent Cohort: 14.35 (1.77)	0/0	ROI	Yes	ASD only
Cheng et al. (2015)	509/418	16.4 (7.08)/ 17.17 (7.97)	85/51	ROI	Yes	ASD/ASP/PDD-NOS
von dem Hagen et al. (2013)	24/15	25 (6)/ 30 (8)	0/0	Seed/ICA	No	HFA (2)/ASP (13)
Hahamy et al. (2015)	73/68	25.82 (0.79)/ 26.6 (0.77)	14/6	Seed	Yes	HFA
Iidaka (2015)	328/312	12.9 (3.0)/ 13.2 (3.1)	61/39	ROI	Yes	ASD
Liu et al. (2020)	548/506	16.86 (7.55)/ 16.59 (8.05)	95/60	Seed	Yes	ASD/ASP/PDD-NOS
Murdaugh et al. (2012)	14/13	22.6 (4.2)/ 21.4 (3.9)	0/0	Seed	No	HFA
Paakki et al. (2010)	27/28	14.49 (1.51)/ 14.58 (1.62)	9/8	ReHo	No	ASD (9)/ASP (19)

*ASP, Asperger’s; HFA, High Functioning Autism; ICA, Independent Component Analysis; PDD-NOS, Pervasive Development Disorder-Not Otherwise Specified; ROI, Region of Interest; ReHo, Regional Homogeneity.*

### Effect Size

#### Schizophrenia

Hedge’s g was used to calculate the effect size of SZ vs. HCs for the primary and secondary FCs depicted in Figure 1 for (1) the original data, (2) GLM, (3) ComBat, and (4) SWD. For the primary FC feature, an ROI corresponding to the MPFC (center of mass MNI coordinates 1.4, 55.9, −7.2; volumes: 193), and ACC (center of mass MNI coordinates 1.6, 33.3, 24.3; volumes: 297) were used for the effect size calculation (Figure 1). For the secondary FC feature (within MPFC FC), two ROIs in the prefrontal cortex were used, MPFC (center of mass MNI coordinates 1.4, 55.9, −7.2; volumes: 193) and MPFC (center of mass MNI coordinates −9.1, 46.4, 40.6; volumes: 206) to calculate the effect size (Figure 1). For the primary FC feature, the effect size decreased compared to the original data for GLM (42.6% decrease), and ComBat (22.5% decrease), and increased for SWD (4.5% increase) (Table 9). For the secondary FC feature (within MPFC FC), the effect size decreased compared to the original data for GLM (40% decrease) and ComBat (23.9% decrease) and increased for SWD (7.9% increase) (Table 9).

**TABLE 9:** Effect size (Hedge’s g) comparison between SZ and HC for the primary FC feature (within MPFC), and for the secondary FC feature (MPFC and ACC) for the original FC data, GLM, ComBat, and SWD.

	**Original**	**GLM**	**%change**	**ComBat**	**%change**	**SWD**	**%change**
Within MPFC	0.3069	0.1761	−42.6%	0.2379	−22.5%	0.3206	4.5%
MPFC—ACC	0.2046	0.1228	−40.0%	0.1557	−23.9%	0.2207	7.9%

#### Autism Spectrum Disorder

Hedge’s g was used to calculate the effect size of ASD vs. HC for the primary and secondary FC features depicted in Figure 2 for (1) the original data, (2) GLM, (3) ComBat, and (4) SWD. For the primary FC feature, a seed region for the MPFC (center of mass MNI coordinates 10.7, 63.0, 10.0; volumes: 202) and PCC/precuneus (center of mass MNI coordinates: 1.5, −52.8, 14.8; volumes: 231) was used for effect size calculation (Figure 2). The effect size decreased compared to the original data for GLM (7.5% decrease) and increased for ComBat (5.1% decrease) and SWD (5.3% increase) for the primary FC feature (Table 10). For the secondary FC feature (frontal pole to temporal lobe FC), a seed region in the frontal pole (center of mass MNI coordinates 1.4, 55.9, −7.2; volumes: 193) and temporal lobe (center of mass MNI coordinates 55.1, −3.6, −25.4; volumes: 201) were used to calculate the effect size (Figure 2). The effect size decreased compared to the original data for GLM (11.4% decrease) and ComBat (1.3% decrease), and increased for SWD (2.9% increase) for the secondary FC feature (Table 10).

**TABLE 10:** Effect size (Hedge’s g) comparison between ASD and HCs for the primary FC feature (MPFC and PCC/precuneus), and for the secondary FC feature (MPFC and MTG) for the original FC data, GLM, ComBat, and the SWD method.

	**Original**	**GLM**	**%change**	**ComBat**	**%change**	**SWD**	**%change**
MPFC–PCC/Precuneus	0.2634	0.2436	−7.5%	0.2769	5.1%	0.2773	5.3%
MPFC—MTG	0.4892	0.4334	−11.4%	0.4829	−1.3%	0.5034	2.9%

*The percent change columns indicate the percent increase/decrease between each method and the original data.*

## Discussion

Previously introduced methods to reduce fMRI site-effects associated with multi-site disorders result in the loss of effect size associated with psychiatric or neurodevelopmental disorders. The SWD method reduced site-effects in large sample sizes in multi-site databases with heterogeneous scan parameters, while improving the effect size of FC features associated with ASD and SZ compared to previous site-effect mitigation methods. This simple method is computationally inexpensive, is applicable to multi-site consortiums post-acquisition, and can be applied to other multi-site fMRI databases.

### Preservation of Functional Networks Associated With Autism Spectrum Disorder and Schizophrenia

ComBat has been proposed to mitigate site-effects in small sizes, when using homogeneous scanning parameters, however it is unknown if it can accurately account for site-effects imposed by heterogeneous scan parameters and whether it can preserve the functional networks associated with psychiatric disorders (Yu et al., 2018). ComBat also centers the FC data of each site to an overall grand mean, thus resulting in harmonized FC features that lose their meaning (Yu et al., 2018; Da-Ano et al., 2020). In addition, GLM may diminish the measurable disease effects when applied to FC data (Yamashita et al., 2019). Therefore, a method is needed to reduce site-effects while maintaining the FC effects present in psychiatric and neurodevelopmental disorders.

Hypoconnectivity in SZ has been widely reported and is associated with symptoms of SZ (Cole et al., 2011; Mwansisya et al., 2013; Du et al., 2016; Fang et al., 2018), while DMN anterior-posterior connectivity in ASD has also been widely reported and has been found to be predictive of clinical symptoms of ASD (Assaf et al., 2010; Weng et al., 2010; Yerys et al., 2015). GLM resulted in a reduction of the effect size of these features by up to 42.6% and ComBat resulted in a reduction of the effect size of these features up to 23.9% in patients vs. control subjects. By de-meaning multi-site FC data, we removed site-effects and improved the effect size by 2.9–7.9% for patients vs. control subjects in the established FC features in both disorders compared to the original data.

The superior performance of SWD compared to GLM may be due to better generalizability and removal of overall site-effects in site de-meaning. In sites with unequal cohort sizes, GLM may introduce a diagnostic group bias. In addition, while diagnostic group is a covariate used in ComBat, the FC values are shifted to an overall mean, which can result in the loss of important diagnostic group information.

### Limitations and Future Work

While there are advantages with SWD, there are several limitations as well. First, while there are many reports of hypoconnectivity in SZ and ASD, there is no conclusive ground truth fMRI neuromarker for either disorder. In addition, while we postulate that this method could be utilized on multiple multi-site databases with various other disorders, this has only been tested on two multi-site consortiums with two different disorders. Therefore, more extensive testing is needed.

## Conclusion

We introduce a site-size demeaning method for reducing site effects in multi-site studies and compared it with two existing methods. The SWD method improved the effect size across these features in two multi-site disorder databases as compared to the original data and previously used harmonization methods (ComBat and GLM).

## Data Availability Statement

Publicly available datasets were analyzed in this study. This data can be found here: Autism data is publicly available from ABIDE (http://fcon_1000.projects.nitrc.org/indi/abide/) and Schizophrenia fMRI data is publicly available from B-SNIP (https://nda.nih.gov/edit_collection.html?id=2274).

## Ethics Statement

This study analyzed two public datasets whose participants were recruited under the ethical guidelines described in the original studies (Tamminga et al., 2013; Di Martino et al., 2014).

## Author Contributions

AR: conceptualization, methodology, software, formal analysis, investigation, visualization, and writing—original draft. KL: methodology, conceptualization, supervision, and writing—review and editing. XH: conceptualization, supervision, and writing—review and editing. All authors contributed to the article and approved the submitted version.

## Conflict of Interest

The authors declare that the research was conducted in the absence of any commercial or financial relationships that could be construed as a potential conflict of interest.

## Publisher’s Note

All claims expressed in this article are solely those of the authors and do not necessarily represent those of their affiliated organizations, or those of the publisher, the editors and the reviewers. Any product that may be evaluated in this article, or claim that may be made by its manufacturer, is not guaranteed or endorsed by the publisher.
